# Bipolar disorders and retinal electrophysiological markers (BiMAR): Study protocol for a comparison of electroretinogram measurements between subjects with bipolar disorder and a healthy control group

**DOI:** 10.3389/fpsyt.2022.960512

**Published:** 2022-09-08

**Authors:** Grégory Gross, Katelyne Tursini, Eliane Albuisson, Karine Angioi-Duprez, Jean-Baptiste Conart, Valérie Louis Dorr, Raymund Schwan, Thomas Schwitzer

**Affiliations:** ^1^Pôle Hospitalo-Universitaire de Psychiatrie d'Adultes et d'addictologie du Grand Nancy, Centre Psychothérapique de Nancy, Laxou, France; ^2^INSERM U1254, Unité d'Imagerie Adaptative Diagnostique et Interventionnelle, Nancy, France; ^3^Faculté de Médecine, Université de Lorraine, Nancy, France; ^4^Fondation FondaMental, Créteil, France; ^5^DRCI, Unité de Méthodologie, Data Management et Statistique UMDS, CHRU de Nancy, Nancy, France; ^6^Département d'Ophtalmologie, CHRU de Nancy, Nancy, France; ^7^UMR CNRS 7039, CRAN Neurosciences, Nancy, France

**Keywords:** bipolar disorder, biomarkers, electroretinogram, electroencephalogram, neuropsychology, actigraphy

## Abstract

**Background:**

Bipolar disorders (BD) is a common, chronic and disabling psychiatric condition. In addition to being characterized by significant clinical heterogeneity, notable disturbances of sleep and cognitive function are frequently observed in all phases of the disease. Currently, there is no readily available biomarker in current clinical practice to help diagnose or predict the disease course. Thus, identification of biomarkers in BD is today a major challenge. In this context, the study of electrophysiological biomarkers based on electroretinogram (ERG) measurements in BD seems highly promising. The BiMAR study aims to compare electrophysiological data measured with ERG between a group of euthymic patients with BD and a group of healthy control subjects. Secondarily, we will also describe the existing potential relationship between clinical, sleep and neuropsychological phenotypes of patients and electrophysiological data.

**Methods:**

The BiMAR study is a comparative and monocentric study carried out at the Expert Center for BD in Nancy, France. In total, 70 euthymic adult patients with BD and 70 healthy control subjects will be recruited. Electrophysiological recordings with ERG and electroencephalogram (EEG) will be performed with a virtual reality headset after a standardized clinical evaluation to all participants. Then, an actigraphic monitoring of 21 consecutive days will be carried out. At the end of this period a neuropsychological evaluation will be performed during a second visit. The primary outcome will be electrophysiological measurements with ERG flash and pattern. Secondary outcomes will be EEG data, sleep settings, clinical and neuropsychological assessments. For patients only, a complementary ancillary study, carried out at the University Hospital of Nancy, will be proposed to assess the retinal structure and microvascularization using Optical Coherence Tomography. Recruitment started in January 2022 and will continue until the end of July 2023.

**Discussion:**

The BiMAR study will contribute to identifying candidate ERG electrophysiological markers for helping the diagnosis of BD and identify subgroups of patients with different clinical profiles. Eventually, this would allow earlier diagnosis and personalized therapeutic interventions.

**Clinical trial registration:**

The study is registered at Clinicaltrials.gov, NCT05161546, on 17 December 2021 (https://clinicaltrials.gov/ct2/show/NCT05161546).

## Introduction

### Background

Bipolar disorder (BD) is a chronic and common psychiatric pathology, which can be particularly disabling. The disease has a global prevalence rate of 1–4% (1), begins at an early age, i.e. predominantly between 15 and 25 years old and persists throughout the life of patients (2). BD is characterized by a recurrence of mood depressive episodes (pathological decrease in mood and energy), hypomanic or manic episodes (pathological increase in mood and energy), or even mixed episodes (simultaneous presence of depressive and manic symptoms). These thymic episodes are interspersed with phases of clinical remission, known as “euthymic” episodes. The disease is associated with a high morbidity and mortality rate and due to the significant functional impact it induces, including during euthymic periods, BD is the cause of poor quality of life and is one of the ten most disabling diseases according to the World Health Organization (3). The diagnosis of BD is mainly clinical and can be supported using scales or questionnaires. The diagnostic delay is estimated at around 10 years. This delay is clearly related to the heterogeneity of the clinical expression of the disease (4). The study of the literature shows that this delay in treatment seriously affects the prognosis, particularly on the functional level, and constitutes a major public health problem (5). In addition, there are no biomarkers, easily usable in current practice, to help the clinical decision for the diagnosis or for predicting the course or prognosis of the disease.

Sleep disturbances and sleep/wake rhythms are major in BD. These disturbances are observed during the different phases of the disease and are major symptoms of mood episodes and belong to the diagnostic criteria for depression, hypomania and mania (6). In addition, these anomalies are also found during euthymic phases (7). Indeed, patients suffering from BD would be more likely to present a more evening chronotype and a more languid and rigid circadian type than healthy subjects as well as a decrease in the efficiency of their sleep, an increase in sleep duration, an increased sleep latency and a prolongation of the duration of awakenings after the onset of sleep (7, 8). These disturbances in sleep and wake/sleep rhythms are associated, among other things, with more frequent relapses, an alteration in quality of life and cognitive disorders (9).

Additionally, neurocognitive deficits are frequently associated with BD. Most typical deteriorations found are an impairment of episodic verbal memory, executive functions, processing speed and sustained attention (10). These troubles can be present during mood episodes but also in around 30% of patients during euthymic phases (11). Cognitive deficits of patients with BD have a direct impact on their psychosocial functioning, on the risk of relapse, on treatment adherence or even on their ability to insight (12). Their early detection associated with the identification of prognostic and predictive biomarkers of the response to cognitive and functional remediation tools is essential in order to be able to offer early and appropriate treatment.

Due to its embryological origin, the retina is an integral part of the central nervous system. The retina is a complex neural tissue, consisting of several layers of retinal neurons. They have structural and functional properties similar to cerebral neurons (13). In addition, molecules involved in brain neurotransmission such as dopamine, serotonin, glutamate or GABA, are also involved in retinal neurotransmission (14). The retina is now considered as a relevant candidate for the study of neurotransmission abnormalities in neuropsychiatric pathologies (15). The function of retinal neurons can be measured using the electroretinogram (ERG). ERG is a simple, fast, inexpensive and non-invasive process which aims to measure the electrical activity of retinal neurons in response to light stimulation and provides indicators of synaptic transmission. This technique can represent an important electrophysiological measurement in biomarker research within psychiatric diseases (16). However, studies evaluating retinal function with ERG in BD are very few (15). In addition to the electrophysiological anomalies mentioned above, structural anomalies at the retinal level have been described. Indeed, several studies carried out in optical coherence tomography (OCT) have shown that subjects with BD present a thinning of the layers of retinal neurons (17). Additionally, results also seem to suggest that retinal thinning may be related to disease progression (18).

As previously described, retinal neurons and cortical neurons have similar properties. It should be noted that measurements of retinal function with ERG and measurements of cortical function with visual evoked potentials (VEP) share the same characteristics. Indeed, both can be performed using light flash stimulation and using luminous black and white reversing checkerboard (pattern stimulation) (13). In addition, they are complementary measures for the analysis of dysfunctions of the central visual pathways (19). Thus, the combined study of VEP using EEG could be relevant and complementary to ERG for a study of all visual neural pathways in neuropsychiatric pathologies.

In this context, highlighting biomarkers in BD is now a major challenge for neuroscience research. Thus, performing electrophysiological measurements provides a unique opportunity to objectively study neurophysiology and a number of electrophysiological parameters altered in BD, could constitute markers of the disease, allowing earlier diagnosis and a guide in medical management.

### Study aims and hypotheses

The aim of the BiMAR study is to analyse the differences in the electrophysiological data measured with ERG pattern (PERG) and flash (fERG) between a group of subjects with BD in euthymic phase and a group of healthy control subjects.

Secondarily, we also want to:

- Compare combined electrophysiological measurements with ERG and EEG between the two groups.
- Identify relations between clinical, neuropsychological and circadian phenotypes in patients with BD and electrophysiological measurements measured with ERG and EEG.

Our main hypothesis is that differences exist in the ERG and EEG measurements between subjects with BD and healthy subjects. Those differences could be identified as candidate markers for BD which, if confirmed in later studies, could be used in current practice to guide the management of patients with BD.

## Methods

### Study design

The BiMAR study is an exploratory, open-label and non-randomized comparative moncentric study applied in psychiatry and neuroscience. This research included two groups of adult subjects: a group of patients with BD in the euthymic phase and a group of healthy control subjects. No blinding procedure is planned in this research.

The BiMAR study protocol was reviewed and approved by the French ethics committee (Comité de Protection des Personnes, Ile de France IV) under the reference 2021/60. The study is also registered at Clinicaltrials.gov under the number NCT05161546. All participants in this research will give their written informed consent before the start of any clinical interview, assessment or measurement.

### Setting and participants

This study will include two groups of participants. A group of euthymic patients with BD and a group of healthy control subjects. Patients eligible for the study will be recruited from the outpatient population followed at the Expert Center for BD in Nancy (France) or followed on an outpatient basis in consultation services of the Psychotherapeutic Center of Nancy. Healthy subjects eligible for the study will be recruited locally from the general population, after a call for volunteers using posters and flyers. A first telephone contact, at least 15 days before the first visit, with interested participants will allow them to present the study in more detail, to verify the main eligibility criteria and to set an appointment for the inclusion visit.

### Inclusion and exclusion criteria

Inclusion criteria for patients were as follows: (1) been diagnosed with BD according to DSM-IV diagnostic criteria using the Mini International Neuropsychiatric Interview (MINI) (20); (2) been currently euthymic for at least 3 months prior to the study, as defined by a score below 10 on the Montgomery-Asberg Depression Rating Scale (MADRS) which assesses depression (21) and by a score below 8 on the Young Mania Rating Scale (YMRS) which assesses mania (22); (3) age 18 or more. Healthy volunteers must not suffer from a personal psychiatric pathology verified with the MINI and be at least 18 years old (20).

Non-inclusion criteria for all participants (patients and healthy subjects) are suffering from psychiatric pathology or substance use disorders according to DSM-IV criteria measured with the MINI (20), excluding BD for the patient group; suffering from neurological or retinal pathology; having a shift work or a jet-lag in the last 15 days; criteria incompatible with the use of the virtual reality headset (Retinaute^®^, BioSerenity) like having an allergy to one of the components of the textile. We will also not include persons treated by sismotherapy during the past year, persons with an uncorrected visual impairment or disabling hearing impairment that does not allow neuropsychological tests to be performed. Finally, pregnant or breastfeeding women will also be excluded as well as subjects with an intellectual disability making it difficult to participate in the study or to understand and follow informations provided to them, adults legally protected, subjects already participating in another interventional trial.

### Procedure

#### Inclusion visit

**Figure 1:**
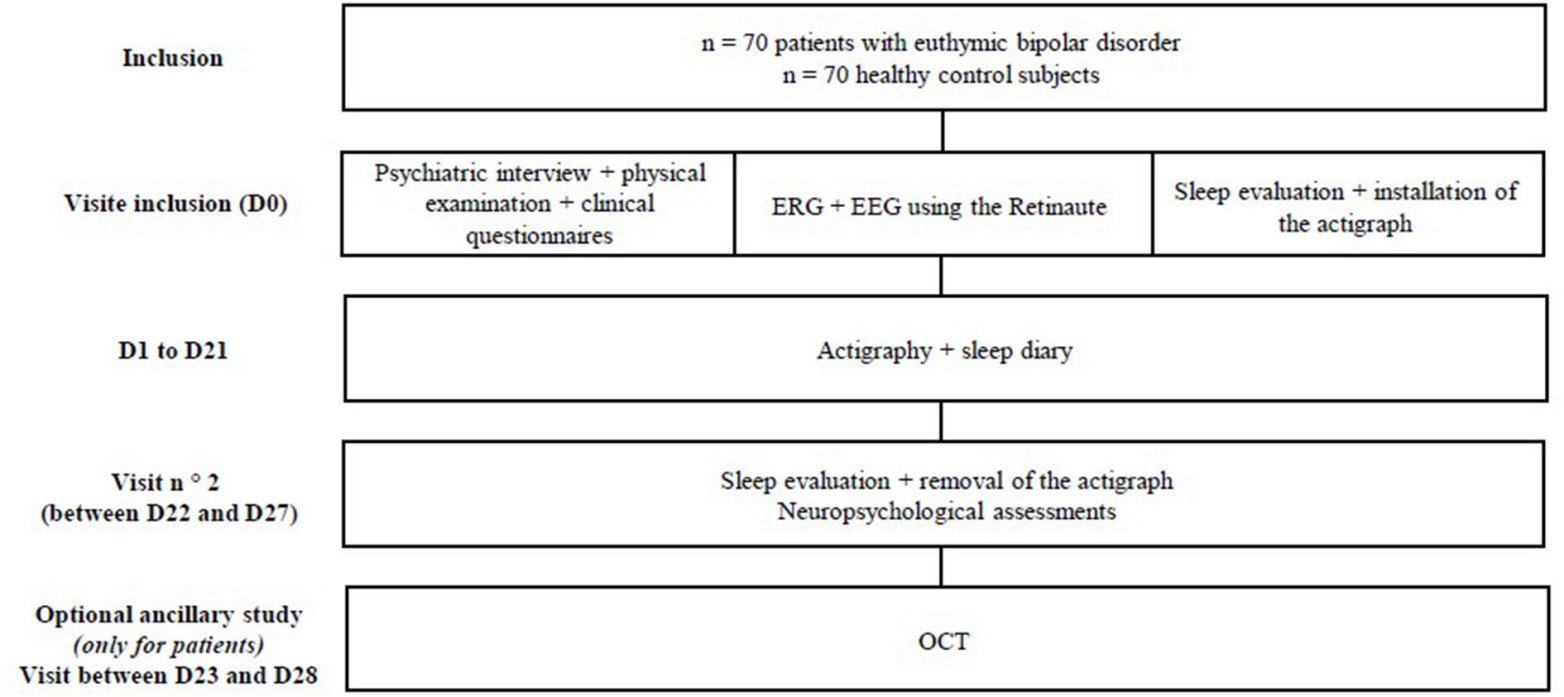
Flowdiagram. D, Day; EEG, Electroencephalogram; ERG, Electroretinogram; OCT, Optical Coherence Tomography.

See Figure 1 for a flow diagram of the BiMAR study and following sections for details of interventions and evaluations. During the inclusion visit and before any examination or act specific to the study, all participants will be informed about objectives and progress of the study and their free, informed and written consent will be obtained. Then, an interview and a clinical examination (including the measurement of height, weight and neck circumference, measurement of visual acuity) will be carried out in order to collect socio-demographic (Date of birth, gender, education level, current occupation) and clinical data (medical history, search for addictive behavior, treatment). The mood will be assessed using MADRS and YMRS (21, 22). In women of childbearing age, a urine pregnancy test will be performed to verify the absence of a current pregnancy. The clinical assessment is based on an interview of approximately 3 h for patients with BD in order to characterize the disorders. This interview will be conducted by a trained psychiatrist or clinical psychologist. The clinical evaluation of patients will be carried out as part of a follow-up visit to the Expert Center for BD in Nancy. Clinical self-questionnaires evaluating “trait” and “state” markers of the disease will also be submitted to patients. For healthy subjects, the interview will be shorter and last an average of 1 h. It will be used to verify inclusion criteria and to eliminate exclusion criteria, in particular the absence of psychiatric disorder. In a second step, all participants will perform an ERG and EEG recording, made with a virtual reality headset and 4 EEG channels (Retinaute^®^, BioSerenity). The duration of this exam is approximately 1 h. The visit will end with an evaluation of sleep of all participants by questionnaires then by the installation of an actigraph (MotionWatch8^®^, CamNtech). The duration of the initial visit will be 1 day for the group of patients and ½ day for the group of healthy control subjects.

#### Between the inclusion visit and the second visit

All participants will wear an actigraph for 21 consecutive days and nights. It measures arm movements in order to assess the quality and quantity of sleep as well as the sleep/wake rhythm. This will be done under the usual living conditions of all participants. They will also complete a sleep diary daily, in the morning after waking up and in the evening before going to bed.

#### Second visit

After the 21 days of recording in actigraphy, a second visit will be offered between day 22 and day 27. First, the actigraph will be removed and a new self-administered questionnaire assessment of sleep and circadian rhythms will be performed. All participants should also bring back the completed sleep diary. Secondly, mood will again be assessed using MADRS and YMRS in order to ensure that all participants are euthymic and neuropsychological evaluations will be carried out with a trained neuropsychologist to assess cognitive function (21, 22). These evaluations will last approximately 2:30 h. The duration of the second visit will be ½ day for all participants. At the end of the second visit, participation in this study (main study) will be over.

#### Optional ancillary study

Patients with BD participating in the study will be offered to participate in an optional ancillary study. They will be informed during the initial visit about the optional ancillary study by the investigator. If they agree to participate, the investigator will obtain informed and written consent on a specific form for this ancillary study. The aim of the ancillary study will be to assess the impact of BD on the retinal structure and microvasculature using Spectral Domain Optical Coherence Tomography (SD-OCT) and OCT-Angiography (OCT-A). SD-OCT and OCT-A are non-invasive imaging techiques that allow to obtain, in a few minutes, cross-sectional images of the retina and an analysis of its vascularity. This will allow us to measure in particular the central macular thickness, the foveal avascular zone area in the superficial capillary plexus, as well as the vessel density of global superficial capillary plexus, deep capillary plexus and choriocapillaris plexus. These examinations will be carried out between day 23 and day 28 (after the second visit) at the Regional University Hospital of Nancy in the ophthalmology department by an experienced ophthalmologist and will last about 10 min.

### Devices

#### Retinaute^®^ (BioSerenity)

The Retinaute^®^ is a portable medical device developed by BioSerenity, France, for performing an ERG. It comes in the form of a virtual reality headset, which can be used in outpatient facilities. It is non-invasive and uses skin electrodes for the collection of parameters. Electrodes will be positioned on the lower eyelid, on the temple and on the forehead, 1 cm above the eyebrows, according to the recommendations of the developer (BioSerenity). fERG allows to study the retina in a global way thanks to a light flash delivered over the entire surface of the retina. The two main analysis parameters are the a-wave and the b-wave with two main components: the amplitude measured in microvolts and the implicit time measured in milliseconds. This allows the study of photoreceptors with the a-wave and bipolar cells with the b-wave. The Retinaute^®^ also allows the realization of a PERG to precisely study the macular area of the retina. The two main parameters are the P50 and N95 waves with two main components: the amplitude measured in microvolts and the implicit time measured in milliseconds. The P50 wave allows the study of the retinal first two layers (photoreceptors and bipolar cells). The N95 wave specifically studies ganglion cells.

PERG and fERG are performed according to the standards of the International Society for Clinical Electrophysiology of Vision (ISCEV) (23, 24). For the PERG measurements, the stimulus is a black and white contrast reversible checkerboard, with 0.8° check size, 93.3% contrast level, 100 cd/m^2^ constant luminance white area, and 4 reversals per second. Dark-adapted 0.01 fERG, dark-adapted 3.0 fERG and dark-adapted oscillatory potentials, are performed after 20 min of dark adaptation. The stimulus in each case was a flash with a strength of 0.01 and 3.0 candela^*^s/m^2^, respectively. Oscillatory potentials are recorded to the 3 candela^*^s/m^2^ flash stimuli. Then, after 10 min of light adaptation with a light background set at 30 cd/m^2^, light-adapted 3.0 fERG and light-adapted 3.0 flicker fERG are recorded. The stimulus is a flash with a strength of 3.0 candela^*^s/m^2^. These signals will be supplemented with 4 EEG channels, *via* cup electrodes applied to the skull and allowing the concomitant performance of an EEG.

Patients will keep their ocular correction for the pattern protocol. They will then remove their correction for the flash protocols. Visual acuity well be measured before the ERG using the Monoyer scale. Averaged retinal responses will first be obtained for each eye, then the parameters (implicit time and amplitude) will be averaged over both eyes for analysis. It is this average result that will be used for the statistical analyses. The examination is painless, non-invasive and takes ~1 h.

#### Actigraphy

Actigraphy is an ecological and non-invasive method allowing a reliable characterization of the sleep/wake cycle. It is a portable system for continuously measuring the motor activity of an individual and appreciating the alternation of activity periods (wakefulness) and rest periods (sleep). An actigraph (MotionWatch8^®^, CamNtech) looks like a wristwatch that will be worn continuously, by convention on the wrist of the non-dominant hand, over periods ranging from several days to several weeks (here 21 days). The actigraph has a button that subjects are invited to press at bedtime and when they wake up, indicating visual markers that will be used during signal analysis (MotionWare^®^ software, CamNtech). This technique is considered the most relevant of the objective, non-invasive measures of sleep disturbances and circadian rhythms currently available and is recommended by the American Academy of Sleep Medicine (AASM) in the study of the sleep/wake cycle. All participants will be able to continue all the usual activities except sea bathing. Actigraphy does not present a known risk.

#### Optical coherence tomography

The SD-OCT is a modern ocular imaging process using infrared radiation and allowing to obtain in a few seconds, and in a non-invasive way, images in section of the eye. It thus generates images of the retina, the optic nerve, the cornea, the anterior chamber, and the iridocorneal angle. The OCT-A module increments on the OCT. It can detect the movement of the blood elements from sequential SD-OCT slices taken at the same location of the retina and obtain a map of the retinal and choroidal vessels, without injection of fluorescent dye. The device used for both exams will be the OCT spectral RS 3,000 Advance 2 + Angioscan (NIDEK, Gamagori, Japan). The images obtained are displayed on a screen and then analyzed using the built-in NIDEK software. SD-OCT and OCT-A examinations are fast, painless, non-contact and last less than a minute. There is no particular risk.

### Measures and outcomes

#### Primary outcomes: ERG measurements

An electrophysiological measurement with fERG and PERG will be carried out to all participants using the Retinaute^®^ device (BioSerenity). The fERG allows the retina to be studied globally. The main analysis parameters recorded will be the amplitude (microvolts) and implicit time (milliseconds) of the a-wave (cones and rods) and the b-wave (bipolar cells). The PERG is used to study the macular area of the retina. Main parameters recorded will be the amplitude (microvolts) and the implicit time (milliseconds) of the P50 (photoreceptors and bipolar cells) and N95 (ganglion cells) waves.

#### Secondary outcomes

##### EEG measurements

With using 4 EEG electrodes connected to the Retinaute^®^ (BioSerenity), we will measure the latency and amplitude of the P100, N170 and N200 waves.

##### Sleep and activity settings

###### Self-assessment questionnaires

Subjective quality of sleep will be assessed by the Pittsburgh Sleep Quality Index (PSQI) (25) which is a 19-item self-assessment questionnaire that investigates the subjective sleep quality during the past month. The daytime sleepiness and severity of insomnia will be evaluated using respectively the Epworth sleepiness scale (ESS) and the Insomnia Severity Index (ISI) (26, 27).

All participants will also complete circadian self-administered questionnaires such as the Horne and Ostberg circadian typology questionnaire, the Composite Scale of Morningness (CSM) and Circadian Type Inventory (CTI) which characterize the phase, amplitude and stability of sleep/wake rhythms (28–30). Finally, the Berlin Questionnaire will be used to estimate the risk of Obstructive Sleep Apnea syndrome (OSA) (31).

###### Actigraphy and sleep diary

All participants will continuously wear on the wrist of their non-dominant hand for 21 consecutive days and nights an actigraph to characterize the sleep/wake cycles and estimate the quantity and quality of sleep. The main sleep parameters measured will be: sleep duration, sleep latency, Wake After Sleep Onset (WASO), sleep efficiency, Fragmentation Index (FI). The main activity parameters recorded will be: Inter-daily Stability (IS), Intra-daily Variability (IV), the relative amplitude of activity and the L5 onset and the M10 onset which corresponds to the onset time of the 5 hours least active and the 10 most active hours during the 24-h cycle. Concomitantly with actigraphy, all participants will complete a sleep diary during these 21 days on which they will register the time they turned off the light to go to sleep, their subjective estimates of sleep latency and the time they got up the next morning.

##### Neuropsychological assessments

The purpose of this assessment is to establish a cognitive profile for each participant. It uses a series of tests widely described in the literature and commonly used today. Several tests would evaluate cognitive functions, including an assessment of verbal episodic memory (California Verbal Learning Test), usually impaired in BD (32). Other cognitive functions such as executive functions would be evaluated thanks to the Test of Attentional Performance (TAP) through the working memory, flexibility and incompatibility subtests (33). In order to complete the executive assessment, the verbal fluency test would be provided (34). Sustained attention is frequently impaired in euthymic BD and will be investigated with the Conners' Continuous Performance Test 3rd Edition (CPT-III) (35). Since aging subjects could be included in the study, the Montreal Cognitive Assessment (MoCA) will be dispensed to detect the presence of mild cognitive impairment (36). Finally, a visual perception test using the Visual Object and Spatial Perception (VOSP) will be performed to inspect visual cognition (37).

##### Clinical assessments

The aim of the clinical evaluation is to characterize the disease in the group of patients. We will therefore look for: family psychiatric history, lifetime comorbidities, number and characteristics of manic, hypomanic and depressive episodes, the age of the first mood episode, the polarity of the first episode and the dominant polarity, the presence of a history suicide attempt, the presence of rapid cycling, mood episodes induced by treatments. We will also characterize the type of BD and research prescribed psychotropic treatments and assess their tolerance and drug response.

#### Other outcomes

As part of the usual management of patients with BD at the Expert Center for BD in Nancy, self-questionnaires evaluating “trait” and “state” markers of the disease will be submitted to patients. Data collected in these questionnaires will perhaps make it possible to identify relations with electrophysiological markers. The “state” questionnaires include an assessment of mood with the Quick Inventory of Depressive Symptomatology self-report (QIDS-SR) and the Altman Self-Rating Mania Scale (ALTMAN), anxiety will be assessed by the State Trait Inventory Anxiety (STAI-A), emotional reactivity will be assessed by the Multidimensional Assessment of Thymic States (MATHYS) and tolerance and adherence to treatments will be assessed by the Patient Rated Inventory of Side Effects (PRISE-M) and the Medication Adherence Rating (MARS) (38–43). Patients treated with lithium will complete the Alda Scale to assess response to treatment (44). The patient's functioning will be assessed using the Functioning Assessment Short Test (FAST) and the Clinical Global Impression Scale (CGI), allowing an assessment of symptom severity and response to treatment (45, 46).

The “trait” questionnaires include an assessment of intensity and emotional lability assessed by the Affective Instability Measure (AIM) and the Affective Lability Scale (ALS), impulsivity and hostility by the Buss-Durkee Hostility Inventory (BDHI) and the Barrat Impulsivity Scale (BIS-10), the search for an antecedent of Attention Deficit/Hyperactivity Disorder will be done using the Wender Utah Rating Scale (WURS) and finally, the search for trauma in childhood will be carried out with the Childhood Trauma Questionnaire (CTQ) (47–52).

Finally, all participants will complete the Fagerström Test to evaluate tobacco dependence and the Edinburgh Handedness Inventory (EHI), which assesses manual preference for determining the wearing side of the actigraph (53, 54).

#### Ancillary study outcomes

This evaluation will only be offered to patients with BD who have given their consent for the ancillary study during a specific visit organized between day 23 and day 28 (after visit n ° 2). SD-OCT will measure central retinal thickness as well as nerve fiber and ganglion cell layer thickness. OCT-A will measure the size of the central avascular zone as well as measure the fractal dimension and vascular density and skeletal density of the global superficial capillary plexus, deep capillary plexus and choriocapillaris plexus.

**Figure 2:**
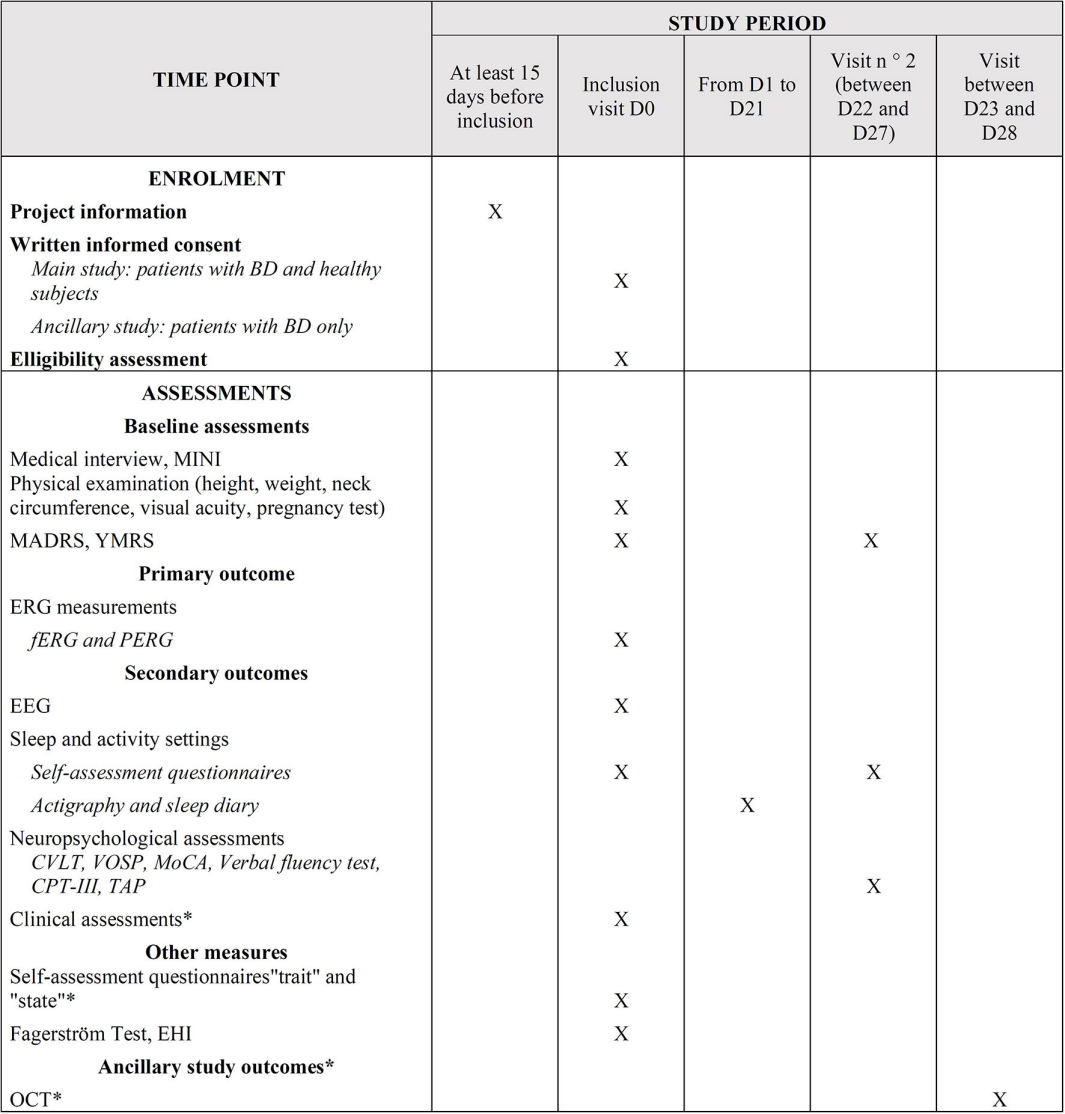
Schedule of enrolment, interventions and assessments. *, Only concerns patients; BD, Bipolar Disorder; CPT, Conners' Continuous Performance Test; CVLT, California Verbal Learning Test; D, Day; EEG, Electroencephalogram; EHI, Edinburgh Handedness Inventory; ERG, Electroretinogram; fERG, electroretinogram flash; MADRS, Montgomery and Asberg Depression Rating Scale; MINI, Mini International Neuropsychiatric Interview; MoCA, Montreal Cognitive Assessment; OCT, Optical Coherence Tomography; PERG, electroretinogram pattern; TAP, Test of Attentional Performance; VOSP, Visual Object and Spatial Perception; YMRS, Young Mania Rating Scale.

All measurements performed during this study are summarized in Figure 2.

### Sample size

Since 2010, analysis of the literature, by synthesizing the results of comparable studies, shows that the ratio between the variability and the clinical difference between healthy subjects and subjects with similar pathologies is generally around 0.5 (for delta/standard deviation or around 2 for standard deviation/delta). With an alpha risk of 5% and a statistical power of 80%, such an effect size leads to a number of 64 subjects per group. With 10% lost to follow-up and/or non-exploitable data, the number of subjects to be recruited in each group is 70. From these elements we therefore set the number of participants to be recruited at 140 subjects: 70 subjects in the patient group and 70 subjects in the healthy control group.

### Statistical analysis

Both descriptive and comparative analyses will be conducted by accounting for the nature and distribution of the variables. Qualitative variables will be described with frequencies and percentage; quantitative variables with the mean ± SD (standard deviation) or with the median (Inter Quartile Range). For quantitative variables, the Student's *t*-test or the Mann-Whitney U test will be used and for qualitative variables, the chi-square test with, if necessary, the exact calculation of Fisher. The Pearson's coefficient or the Spearman's rho will allow analysis of the correlations. A multivariate analysis will be conducted according to the results of univariate analysis. Regarding the analysis of ERG parameters, after checking the distribution, these will be analyzed *via* generalized estimating equations (GEE) in case of gamma distribution. This is a robust statistical method relevant in the analysis of repeated measures with non-normal response variables (55). Finally, a receiver operating characteristic curve (RoC) will be generated for variables that differed between groups in order to dichotomize the variables of interest and calculate the associated sensitivity and specificity values.

An intermediate data analysis is planned 9 months (mid-term study) after the start of inclusions. The statistical significance level is set at 1% for the interim analysis and 4% for the final analysis. IBM™ SPSS Statistics v23 will be used for the data analysis.

### Data management

Data collection and management will be carried out according to French law. Study data will be collected during the various visits by the investigator, or any designated person, directly into a research-specific observation notebook for each participant. All information collected as part of this study will be pseudonymized. The data are then entered into a database dedicated to the study, hosted on the secure internal network of the Psychotherapeutic Center of Nancy and accessible only to investigators. At the end of the study, all paper documents are archived and then kept for 15 years. At the end of the study and after analysis of the data relating to this research, all participants can be informed of the overall results. Data presented in publications will be completely anonymous.

### Safety monitoring

The participation in the BiMAR study does not involve any particular risk expected. Indeed, this study includes only safe tests and evaluations. Therefore, no research-specific event or adverse effect is expected. The investigators have a good experience in the use of the different devices. Subjects with epilepsy will be excluded from the study because of the repeated light stimulation with the Retinaute^®^ (BioSerenity). We do not expect an effect of the study on the course of BD for patients. However, the natural course of BD may expose patients to the occurrence of an acute decompensation of the illness. In this case, their participation will be stopped and an adapted treatment will be proposed. The investigators will systematically question all participants during various visits in order to look for possible adverse events. The presence or absence of adverse events will be recorded in the study's case report form at each study visit. If an adverse event has occurred from the date of inclusion and throughout the duration of the study, it will be declared without delay according to the French usual reporting's procedure to the concerned health vigilance institution. Anyone with an adverse event will receive treatment appropriate to their condition and will be followed until the event is resolved or until the end of the research. If necessary, the experienced device or test will be stopped for that person.

### Duration of the study and stopping rules

All participants are volunteers and have given their written consent after receiving information on the objectives and progress of the study. All participants are informed that they are free to accept or decline to participate in this study, just as they are free to terminate their participation at any time during the research. This will not influence the quality of care that will be provided to patients and will not affect their medical care. Note that evaluations do not require interrupting or modifying treatments in progress. The BiMAR study consists of two visits and a 21-day outpatient period. At the end of the second visit, participation in this study will be terminated except for patients who have agreed to participate in the ancillary study who will benefit from a third visit. Given the nature of our research, no particular follow-up is necessary at the end of the participation. Any participant can stop participating in the study at any time and for any reason. The investigator may temporarily or permanently discontinue an individual's participation in this research for any reason which could affect patient safety or would be in the best interests of the patient. In the event of drop-out, the participant is replaced by another of the same category (patient vs. healthy subject).

## Study status

Recruitment of participants began in January 2022 and will continue over a period of 18 months until July 2023. The latest data collection is scheduled for the end of July 2023, after the last visit of the last included participant. We plan to disseminate our findings through oral and poster presentations at national and international conferences, as well as through international publications.

## Discussion

The aim of the BiMAR study is to compare electrophysiological data measured with ERG between subjects with BD in the euthymic phase and healthy control subjects. Secondarily, we will also study the existing potential relationship between clinical, circadian and neuropsychological phenotypes of patients and electrophysiological data measured with EEG and ERG. To our knowledge, this study will be the first one with these objectives and with a specific methodology in a population of patients with BD.

We hypothesize that electrophysiological data measured with ERG associated with EEG data could help to identify candidate markers. These will then need to be confirmed in subsequent studies with larger samples. If they are validated they could be used in current practice in order to improve the diagnosis and management of patients. Thus, these measures could be integrated in the course of care of patients with BD and contribute to personalized precision psychiatry. One other feature of this research is to use a portable ERG device (Retinaute^®^, BioSerenity), the usability and distribution of which on an outpatient basis would allow good accessibility.

The identification of biomarkers in BD represents a major diagnostic and prognostic challenge in psychiatry. Thus, electrophysiological measurements, in particular with ERG, could be candidate markers of interest in order to be able to improve the diagnosis of patients suffering from BD and in the identification of patient clusters. This will make it possible to improve the diagnosis and to offer earlier and more appropriate therapeutic interventions, thereby reducing the functional impact and the morbidity and mortality of BD.

Regarding the recruitment of healthy control subjects, the population base of the city of Nancy should allow us without much difficulty. For patients, we rely on the active file of the Expert Center for BD in Nancy and on the outpatient consultations of the Psychotherapeutic Center of Nancy. As this research does not offer long-term follow-up, we expect a low rate of drop out or lost to follow-up. The different interventions should have good acceptability since they are based exclusively on non-invasive devices. In addition, the two visits and all the interventions will be carried out on a single site at the Psychotherapeutic Center of Nancy or at home for recording by actigraphy. Only the ancillary study offered exclusively to patients will take place in a different place, at the Regional University Hospital of Nancy.

Evaluations, interventions, and measurements carried out in this research, such as electrophysiological parameters of ERG, EEG, actigraphy and OCT, are done using routine, inexpensive, non-invasive, painless and very well tolerated examinations. Thus, this study does not represent any particular risk. Moreover, it will not cause any change in the care and treatment of patients.

Certain limitations inherent in the interventions are known and described. They will be considered in this study. Indeed, several conditions may be associated with changes in the function and/or retinal structures. We can cite certain medical comorbidities such as obesity, diabetes, OSA, the consumption of certain toxic substances including tobacco, as well as the taking of certain medications (15). However, these comorbidities are frequently associated with BD and the disease requires long-term treatment. We know that these factors can have an impact on the ERG signal or even EEG. This is the reason why we will take it into account when processing and analyzing the data. Recording in actigraphy requires all participants to remember to press the button on the watch when going and getting out of bed. It may nevertheless be compensated through the sleep diary that will correct missing data. Also note that participants with time awake during the night by staying completely still could be counted as sleep by the device and thus overestimate sleep duration. We know that performing neuropsychological tests can require a significant attentional load, especially for patients who may have residual cognitive impairment associated with BD. This will be offset by the organization of breaks during the execution of the tests.

The ancillary study will consist of carrying out an OCT examination for the group of patients. This complementary module will allow the study of retinal structural changes within a significant sample with regard to data from the literature (56). We will also carry out a study of the retinal microvasculature, for which there is no data in the literature, to our knowledge, in patients with BD.

Finally, one of the specificities and strengths of the BiMAR study is to concomitantly combine the study of several electrophysiological markers and to associate a search for a relationship with circadian, sleep and neuropsychological markers.

## Data availability statement

All the data collected during this research and the statistical analyzes carried out are available from the corresponding author on reasonable request.

## Ethics statement

The study has been reviewed and approved by the French Agency for the Safety of Medicines and Health Products and by the French ethics committee (Comité de Protection des Personnes, Ile de France IV; protocol number 2021/60). All participants provided their written informed consent to participate in this study.

## Author contributions

TS and GG were the main contributor to the design of the study. KT contributed to the study design. GG, KT, and TS contributed substantially to the writing of the manuscript. RS and VL contributed to revising the study protocol. EA contributed to the conception of the data analysis of the study and the writing of the data analysis section of the manuscript. KA-D and J-BC contributed to the design of the ancillary study and the writing of the parts presenting the OCT and the ancillary study. All authors read and approved the final manuscript.

## Funding

This study is supported by the Psychotherapeutic Center of Nancy and BioSerenity. The funders has not be involved in the design of the study or in the data collection, analysis or interpretation of the data, and nor in writing of the present manuscript.

## Conflict of interest

The authors declare that they have a scientific collaboration with BioSerenity in this study using the Retinaute^®^ (BioSerenity).

## Publisher's note

All claims expressed in this article are solely those of the authors and do not necessarily represent those of their affiliated organizations, or those of the publisher, the editors and the reviewers. Any product that may be evaluated in this article, or claim that may be made by its manufacturer, is not guaranteed or endorsed by the publisher.

## Abbreviations

AASM: American Academy of Sleep Medicine
AIM: Affective Instability Measure
ALS: Affective Lability Scale
ALTMAN: Altman Self-Rating Mania Scale
BD: Bipolar Disorder
BDHI: Buss-Durkee Hostility Inventory
BIS-10: Barrat Impulsivity Scale
CGI: Global Impression Scale
CHRU: Regional University Hospital of Nancy
CPT: Conners&#8217
Continuous Performance Test
CSM: Composite Scale of Morningness
CTI: Circadian Type Inventory
CTQ: Childhood Trauma Questionnaire
CVLT: California Verbal Learning Test
D: Day
DSM: Diagnostic and Statistical Manual of Mental Disorders
EEG: Electroencephalogram
EHI: Edinburgh Handedness Inventory
ERG: Electroretinogram
FAST: Functioning Assessment Short Test
fERG: electroretinogram flash
FI: Fragmentation Index
IS: Inter-daily Stability
ISCEV: International Society for Clinical Electrophysiology of Vision
IV: Intra-daily Variability
MADRS: Montgomery and Asberg Depression Rating Scale
MARS: Medication Adherence Rating
MATHYS: Multidimensional Assessment of Thymic States
MINI: Mini International Neuropsychiatric Interview
MoCA: Montreal Cognitive Assessment
OSA: Obstructive Sleep Apnea syndrome
OCT: Optical Coherence Tomography
OCT-A: Optical Coherence Tomography-Angiography
PERG: Electroretinogram pattern
PRISE-M: Patient Rated Inventory of Side Effects
PSQI: Pittsburgh Sleep Quality Index
QIDS-SR: Quick Inventory of Depressive Symptomatology self-report
STAI-A: State Trait Inventory Anxiety
SD-OCT: Optical Coherence Tomography Spectral Domain
TAP: Test of Attentional Performance
VEP: Visual Evoked Potential
VOSP: Visual Object and Spatial Perception
WASO: Wake After Sleep Onset
WURS: Wender Utah Rating Scale
YMRS: Young Mania Rating Scale.
